# Duodenal biopsy and drainage procedures via the hepaticogastrostomy route using a newly designed device delivery system

**DOI:** 10.1055/a-2547-6052

**Published:** 2025-03-12

**Authors:** Koichi Soga, Takeshi Fujiwara, Fuki Hayakawa, Mayumi Yamaguchi, Ikuhiro Kobori, Masaya Tamano

**Affiliations:** 1 26263 Department of Gastroenterology, Dokkyo Medical University Saitama Medical Center, Koshigaya, Japan

Assessing postoperative retroperitoneal malignant invasion with duodenal stenosis and obstructive jaundice is challenging. After surgical reconstruction, endoscopic access through the retroperitoneum becomes difficult, complicating efforts to improve these conditions. Endoscopic ultrasonography-guided hepaticogastrostomy (EUS-HGS) is a highly effective treatment for obstructive jaundice 1 . However, deploying several devices through the needle tract remains considerably challenging.

This video presents a 61-year-old Japanese man who had undergone distal gastrectomy and Roux-en-Y reconstruction for gastric cancer 4 years prior. Imaging indicated recurrence of gastric cancer, inducing obstructive jaundice with retroperitoneal invasion. Thus, histological diagnosis and biliary drainage were planned using the EUS-HGS procedure ( Fig. 1 ).

**Fig. 1:**
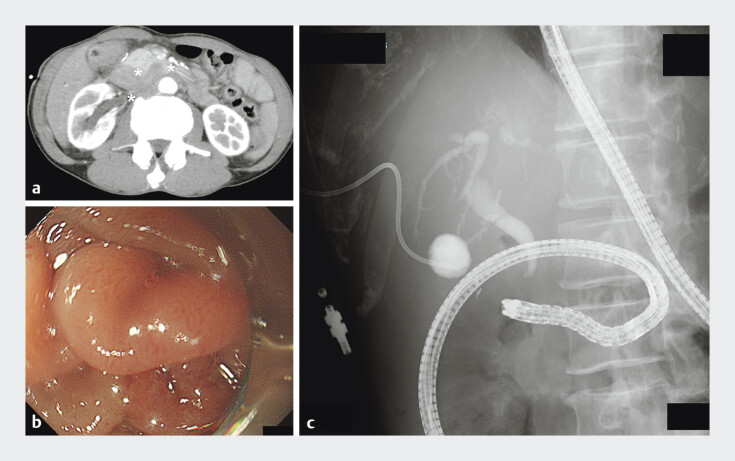
Abdominal imaging. **a** Computed tomography images showed retroperitoneal thickness with duodenal stenosis and obstructive bile duct stenosis. White stars (*) indicate gastric cancer recurrence. **b, c** Endoscopic ( **b** ) and fluoroscopic ( **c** ) images showed that single-balloon enteroscopy could not access the papilla of Vater because of gastric cancer recurrence with retroperitoneal invasion.

EUS-HGS was performed using a convex echoendoscope (GF-UCT260; Olympus Medical Systems, Tokyo, Japan). The bile duct (segment B2) was punctured under EUS guidance using a 19-gauge needle (Ez-shot 3 Plus; Olympus Medical Systems). A 0.025-inch guidewire was inserted, followed by mechanical dilation of the needle tract using a catheter and bile juice aspiration. A guide sheath with a tapered tip (EndoSheather; Piolax Inc., Yokohama, Japan) was inserted over the guidewire. The inner catheter was removed, and the outer sheath remained inside the common bile duct. Biopsies of the duodenal mucosa were performed using biopsy forceps 1.8-mm in diameter (Radial Jaw 4P; Boston Scientific, Marlborough, Massachusetts, USA). Finally, a 5.9-Fr fully covered self-expandable metal stent (6 mm × 12 cm HANAROSTENT; Boston Scientific) was placed. The biopsy confirmed gastric cancer recurrence ( Fig. 2 , Fig. 3 , Video 1 ).

**Fig. 2:**
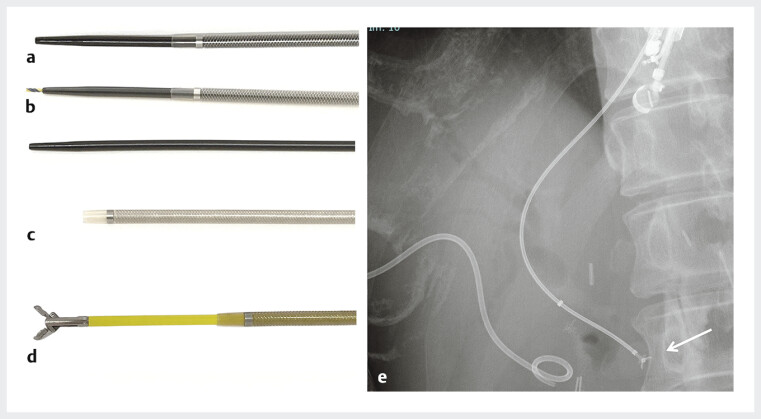
The endoscopic tapered sheath (EndoSheather; Piolax Inc., Yokohama, Japan). **a** Minimal caliber difference exists between inner and outer catheters at the device tip. **b** The gap between the sheath and guidewire is minimized, allowing for smooth insertion of the device into the puncture site. **c** The outer sheath with a mesh-braided structure provides optimal kink resistance. **d** The inner catheter is removed, and the outer sheath remains inside the common bile duct. Biopsy forceps with 1.8-mm diameter (Radial Jaw 4P; Boston Scientific, Marlborough, Massachusetts, USA) is inserted into the outer sheath of the device delivery system. **e** A combination of pushing and pulling the biopsy forceps and opening and closing the tip are performed to guide the forceps toward the target site (white arrow).

**Fig. 3:**
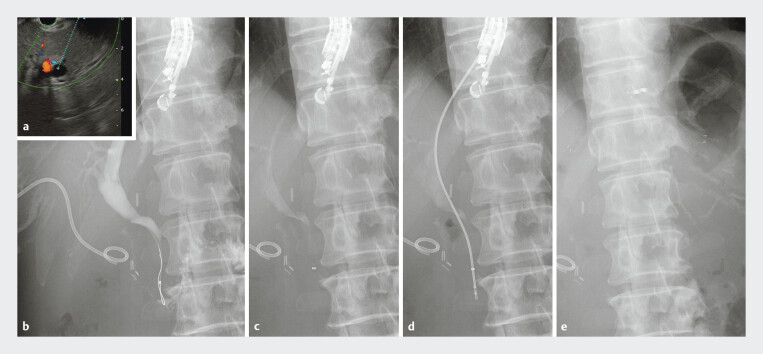
The endoscopic ultrasound-guided hepaticogastrostomy procedure (EUS-HGS) with histological diagnosis and biliary drainage. **a** EUS-HGS was performed using a convex echoendoscope (GF-UCT260; Olympus Medical Systems, Tokyo, Japan). The bile duct (segment B2) was punctured under EUS guidance using a 19-gauge needle (Ez-shot 3 plus; Olympus Medical Systems). **b** A 0.025-inch guidewire was inserted, followed by mechanical dilation of the needle tract using a catheter and bile juice aspiration. A guide sheath with a tapered tip (EndoSheather; Piolax Inc., Yokohama, Japan) was inserted over the guidewire. **c** The inner catheter was removed, and the outer sheath remained inside the common bile duct. **d** Biopsies of the duodenal mucosa were performed using biopsy forceps with 1.8-mm diameter (Radial Jaw 4P; Boston Scientific, Marlborough, Massachusetts, USA). **e** Finally, a 5.9-Fr fully covered self-expandable metal stent (6 mm × 12 cm; HANAROSTENT, Boston Scientific) was placed.

Initial experience with duodenal biopsy and drainage procedures via the hepaticogastrostomy route using a newly designed device delivery system.Video 1

This video demonstrates a biopsy technique using a novel endoscopic sheath via the EUS-HGS route 2 3 . This device functions as a dilation tool and delivery system, enabling mechanical dilation of the needle tract while facilitating the smooth insertion of biopsy forceps through the indwelling outer sheath. The sheath effectively bridges the gap at the target site, minimizing the risk of bile leakage. Consequently, EUS-HGS can be performed promptly.

Endoscopy_UCTN_Code_TTT_1AS_2AH

## Notes

Endoscopy E-Videos https://eref.thieme.de/e-videos
*E-Videos* is an open access online section of the journal *Endoscopy* , reporting on interesting cases and new techniques in gastroenterological endoscopy. All papers include a high-quality video and are published with a Creative Commons CC-BY license. Endoscopy E-Videos qualify for HINARI discounts and waivers and eligibility is automatically checked during the submission process. We grant 100% waivers to articles whose corresponding authors are based in Group A countries and 50% waivers to those who are based in Group B countries as classified by Research4Life (see: https://www.research4life.org/access/eligibility/ ). This section has its own submission website at https://mc.manuscriptcentral.com/e-videos .
